# Cultivar and Postharvest Storage Duration Influence Fruit Quality, Nutritional and Phytochemical Profiles of Soilless-Grown Cantaloupe and Honeydew Melons

**DOI:** 10.3390/plants11162136

**Published:** 2022-08-17

**Authors:** Boitshepo L. Pulela, Martin M. Maboko, Puffy Soundy, Stephen O. Amoo

**Affiliations:** 1 Department of Crop Sciences, Tshwane University of Technology, Private Bag X680, Pretoria 0001, South Africa; 2 Agricultural Research Council, Vegetables, Industrial and Medicinal Plants, Private Bag X293, Pretoria 0001, South Africa; 3 Department of Botany and Plant Biotechnology, University of Johannesburg, P.O. Box 524, Auckland Park, Johannesburg 2006, South Africa; 4 Indigenous Knowledge Systems Centre, Faculty of Natural and Agricultural Sciences, North-West University, Private Bag X2046, Mmabatho 2790, South Africa

**Keywords:** antioxidant, β-carotene *Cucumis melo*, flavonoids, mineral element, vitamin C

## Abstract

There is an increasing demand for sweet melon (*Cucumis melo* L.) fruit in fruit and vegetable markets due to its nutritional content, resulting in different cultivars being grown in different production systems. This study evaluated the nutritional and phytochemical contents of soilless-grown cantaloupe and honeydew sweet melon cultivars at harvest and postharvest. At harvest, vitamin C and β-carotene concentrations were higher in orange-fleshed (cantaloupe) cvs. Magritte, Divine, Majestic, Cyclone, MAB 79001, E25F.00185, E25F.00075 and Adore, compared to green-fleshed (honeydew) cvs. Honey Brew and Honey Star. The zinc (Zn), phosphorus (P), potassium (K), magnesium (Mg) and calcium (Ca) contents were higher in orange-fleshed compared to green-fleshed cultivars. Total phenolics content (TPC) in cv. E25F.00075 was the highest (2.87 mg GAE∙g^−1^ dry weight). A significant, positive, correlation occurred between β-carotene and Zn, P, K, Ca and Mg contents. Postharvest storage duration affected TPC and total soluble solid content. The interaction of cultivar and postharvest storage duration affected flavonoid, vitamin C and β-carotene contents, free radical scavenging activity and fruit juice pH. Vitamin C and β-carotene contents decreased with increased postharvest storage duration while flavonoid content increased. The cantaloupe cultivars performed significantly better compared to the honeydew cultivars as evident in their high mineral element content, and vitamin C and β-carotene concentrations. Selection of appropriate cultivars in a production system should consider variation in nutritional traits of cultivars and postharvest storage duration. Soilless production of sweet melon cultivars in tunnels offers a viable alternative to open field to produce high-quality melons at harvest and postharvest.

## 1. Introduction

Sweet melons (*Cucumis melo* L., family: Cucurbitaceae), commonly known as spanspek or muskmelons, have become a popularly consumed fresh fruit across the world. Although most melons are grown in open fields, commercial production of melons in soilless culture under greenhouse conditions is becoming a preferred option among farmers to improve yield [1,2,3]. Changes in weather conditions and availability of land and suitable soil type coupled with build-up of soil-borne pathogens and fluctuations in available nutrient supply are among the challenges associated with field-based monoculture systems [4]. Protected soilless culture alleviates some of these problems while extending the harvest period with the potential to increase yield compared to production under open field conditions [5].

Improved fruit yield per unit area may be achieved in soilless culture under high-tunnel protection than in a field because plants are arranged more uniformly, large gaps between plants and rows are avoided and light interception is optimized [6]. Soilless culture can produce fruit and vegetables with better taste, color, texture and higher nutritional value than those from soil cultivation [7]. Melon growers should consider soilless culture under protection or greenhouse conditions that can provide a consistent supply of high-quality fruit with low risk of crop failure. Systems such as non-temperature controlled (NTC) plastic tunnels can be employed as they use natural ventilation to reduce temperature [4,8]. However, the use of soilless culture under protection does not automatically result in production of high-quality melons. High yields and quality are dependent on cultivar choice, crop management and growing season [9]. Sweet melon cultivars vary in development and yield in NTC plastic tunnels [10], with no information on nutritional and phytochemical content at harvest and postharvest.

Orange-fleshed cantaloupe and green-fleshed honeydew melons are rich sources of β-carotene, vitamin C (ascorbic acid), vitamin E and folic acid [11,12,13]. Melons have low fat and sodium with no cholesterol [11], making them a good addition to a healthy diet. There is increased consumer awareness regarding melon fruit with good visual and eating quality, health-promoting compounds and properties such as antioxidant capacity. Unlike cooked vegetables in which nutritional values become altered during cooking, the nutritional profiles of both orange- and green-fleshed sweet melon fruit are retained due to being eaten raw.

The increase in demand for sweet melon fruit has resulted in production and release of new sweet melon varieties. Nutritional content can vary among varieties, and the production system used to grow them [14,15,16]. This study evaluated nutritional and phytochemical qualities at harvest and after postharvest storage of fruit from sweet melon cultivars grown in soilless culture.

## 2. Results and Discussion

### 2.1. Effect of Cultivars on Fruit Nutritional and Phytochemical Quality at Harvest

At harvest, fruit mineral element content varied among cultivars (Table 1). Cultivar Magritte had the highest fruit potassium and phosphorus contents. Cultivar Majestic had the highest magnesium and calcium contents, which were 2-fold that of the green-fleshed sweet melons, cvs. Honey Brew and Honey Star. All orange-fleshed cultivars had higher Zn content compared to the green-fleshed cultivars. Selection of orange-fleshed cultivars may be a particularly important consideration in sub-Saharan Africa and South Asia where zinc deficiency is prevalent [17]. Green-fleshed cvs. Honey Brew and Honey Star contained the lowest levels of all mineral elements. Variations in fruit mineral element content might thus be linked to cultivar differences.

**Figure 1:**
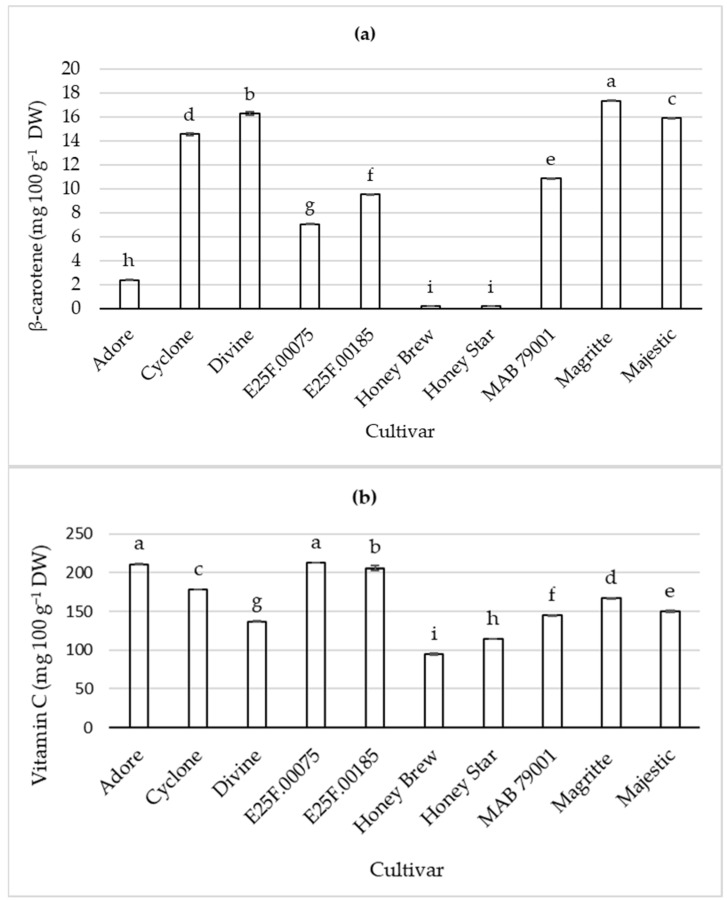
β-Carotene (**a**) and vitamin C (**b**) contents of fruit from sweet melon cultivars at harvest. Bars (with error bars indicating standard error of means) with different letters are significantly different at *p* ≤ 0.05 (Fisher’s protected LSD test, *n* = 4). DW = dry weight.

The β-carotene content in harvested fruit, which is responsible for the orange color pigments in orange-fleshed sweet melon cultivars, was higher, as expected, in orange-fleshed cultivars (Figure 1). Honey Brew and Honey Star cvs. have a pale green color and lack orange color pigments. Thus, the concentration of β-carotene is dependent on the cultivar and fruit flesh color, which is linked to the pigments present in the fruit. Pigment (carotenoid and chlorophyll) compositions are largely different between cantaloupe and honeydew fruits [16]. With some limitations, color may be used as an indicator for β-carotene-rich sweet melon cultivars. Cantaloupe fruits do not contain chlorophyll, and β-carotene accounts for the majority of total carotenoids, while in honeydew melons, the major pigments are chlorophylls a and b [16]. β-ionone is a major norisoprenoid derived from β-carotene with a typical aroma, rarely found or in small quantities when present in green- and white-fleshed melon varieties [18]. Honeydew melons accumulate less β-carotene than cantaloupes [16]. In the current study, there were significant variations in the β-carotene content of the cantaloupe types. Cultivar type, fruit size, growing location and year may interact to influence β-carotene content of orange-fleshed muskmelon fruit [19]. The latter two factors can be excluded in the current study as the fruit were obtained from cultivars grown at the same time and in the same location/production system.

Cultivar also influenced fruit vitamin C content at harvest (Figure 1). All orange-fleshed cultivars had higher vitamin C content compared to the green-fleshed cultivars. The vitamin C contents of orange-fleshed cvs. Adore, E25F.00075 and E25F.00185 were approximately 2-fold that of the green-fleshed cvs. Honey Brew and Honey Star. Variation in fruit color influenced concentration of vitamin C.

**Figure 2:**
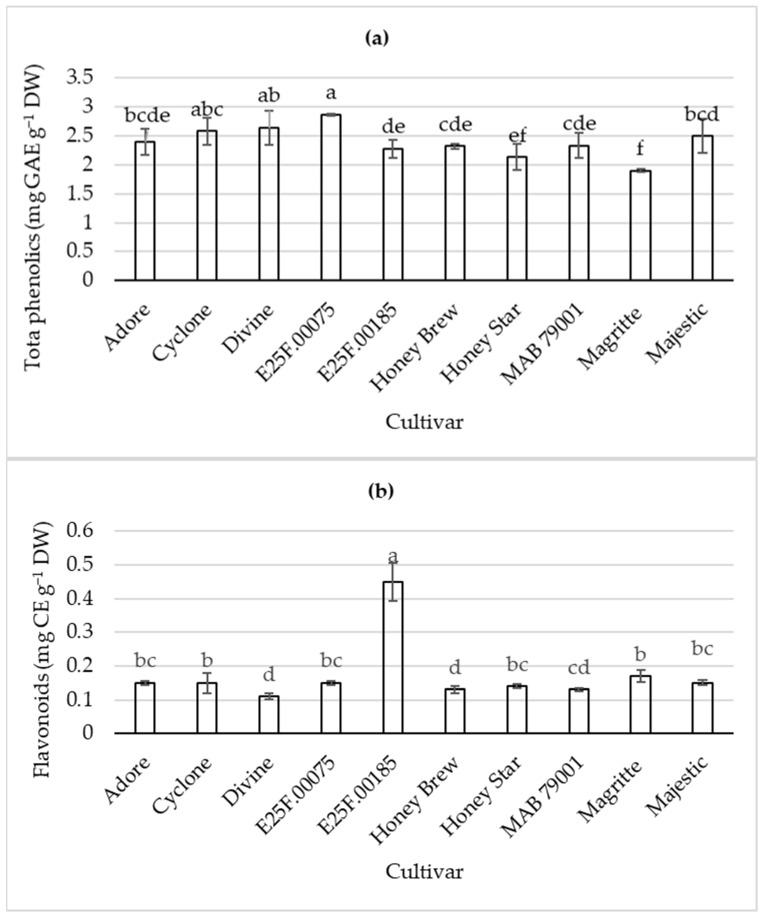
Total phenolics (**a**) and flavonoid (**b**) contents of fruit from sweet melon cultivars at harvest. Bars (with error bars indicating standard error of means) with different letters are significantly different at *p* ≤ 0.05 (Fisher’s protected LSD test, *n* = 4). GAE = gallic acid equivalent; CE = catechin equivalent; DW = dry weight.

Cultivar E25F.00075 exhibited the highest total phenolic content with 51% increased total phenolic content, compared to cv. Magritte with the lowest total phenolic content at harvest (Figure 2). Cultivars E25F.00075 and Divine (both cantaloupe type) exhibited higher total phenolic content than honeydew sweet melon cvs. Honey Brew and Honey Star. Cultivar E25F.00185 (cantaloupe type) had the highest flavonoid content; more than double the flavonoid content in all other cultivars (including the honeydew types) (Figure 2). Different cantaloupe type cultivars had the highest total phenolic and flavonoid contents whereas the honeydew type cultivars were among the cultivars with low total phenolic and flavonoid contents. Thus, fruit color seemed to be associated with β-carotene, vitamin C, flavonoid and total phenolic contents, with the cantaloupe cultivars in this study as more nutrient-rich sweet melons.

**Figure 3:**
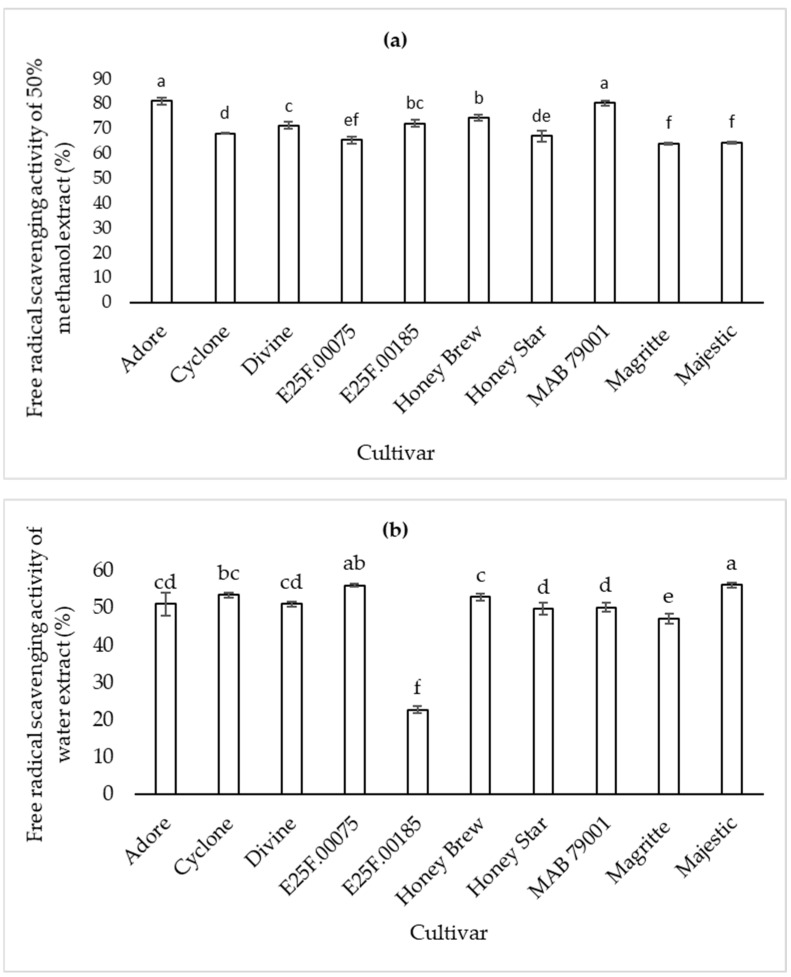
Free radical scavenging activity of 50% methanol (**a**) and water (**b**) extracts of fruit from sweet melon cultivars at harvest. Bars (with error bars indicating standard error of means) with different letters are significantly different at *p* ≤ 0.05 (Fisher’s protected LSD test, *n* = 4).

Higher free radical scavenging activity was recorded with 50% methanol extract for all cultivars compared to their respective water extracts (Figure 3). Water extracts from cvs. Majestic and E25F.00075 (cantaloupe type) had higher free radical scavenging activity than the honeydew melons, cvs. Honey Brew and Honey Star. The 50% methanol extracts of cvs. MAB 79001 and Adore (cantaloupe type) had higher free radical scavenging activity than honeydew melons. The antioxidant amount in muskmelon varied between stages and between cultivars [20]. Cultivar type had a significant influence on the antioxidant activity regardless of the solvent used as both extracts recorded higher activity in the orange-fleshed cantaloupes, therefore cultivar type remains the main determinant factor. Zeb [21] reported higher radical scavenging activity with the aqueous methanolic extracts than individual methanol and water extracts, however, these results were from seeds while the current study compared fruit extracts. The efficiency of extraction, as well as extract yield, depends on type of solvents used, solubility and polarity of the active compounds in the solvent, including time and temperature of extraction [22]. High total antioxidant activity occurs in the early stages of muskmelon fruit ripening with its maximum in the premature stage [23]. Though the antioxidant activity may be at its maximum at the premature stage, sweet melons are rarely harvested at this stage of growth, as this may affect eating quality for local and commercial markets. Sweet melons are harvested at the fully ripe stage when they have attained optimum quality.

The correlation between sweet melon nutritional and phytochemical properties at harvest varied (Table 2). A significant, positive, correlation was established between β-carotene content and all mineral elements, except calcium, with β-carotene and phosphorus having the highest positive correlation. Both phosphorus and zinc contents were individually significantly positively correlated with fruit vitamin C, β-carotene and all other mineral element contents. This relationship may explain why green-fleshed sweet melon cultivars recorded low vitamin C and β-carotene contents as these cultivars had low concentrations of all mineral elements. Nonetheless, a strong positive multivariate correlation is advantageous for direct and indirect selection in biofortification as well as nutritional and phytochemical quality improvement programs. 

**Figure 4:**
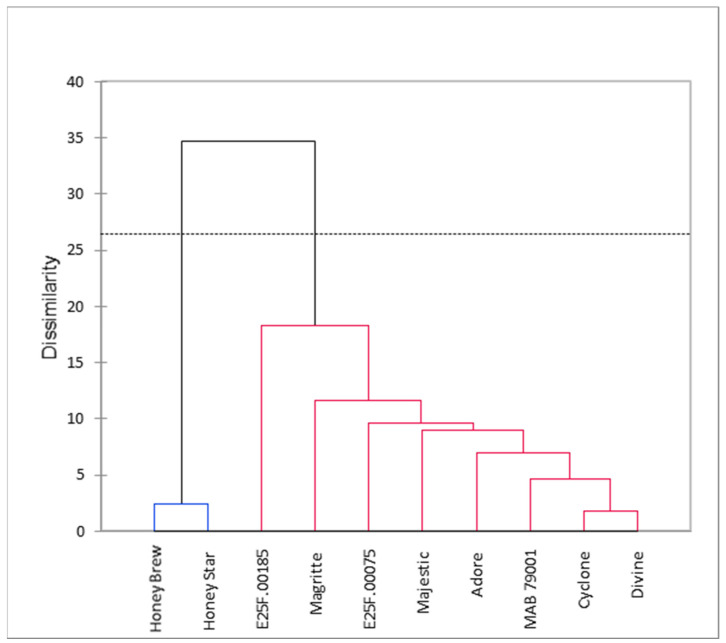
Dendrogram indicating the interrelatedness of the cultivars based on the measured fruit quality at harvest.

**Figure 5:**
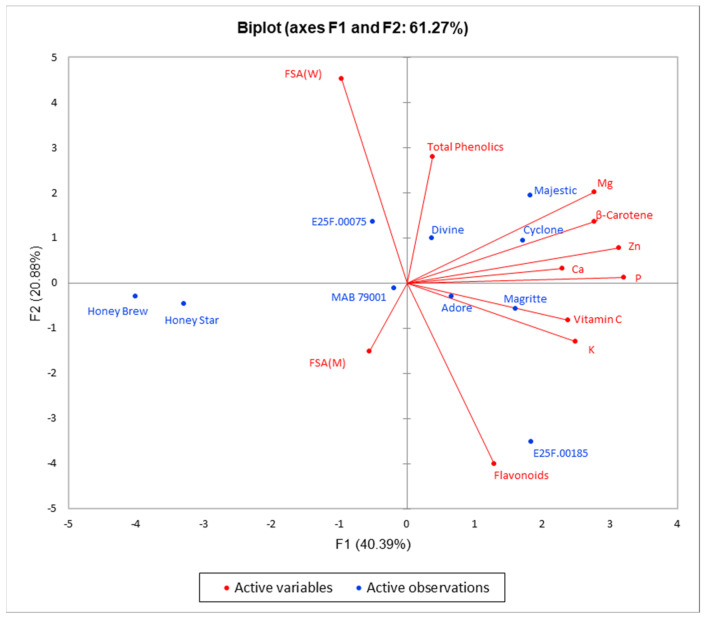
Principal component biplot of fruit nutritional and phytochemical qualities for the evaluated honeydew and cantaloupe melon cultivars at harvest. FSA (W) = free radical scavenging activity of water extract; FSA (M) = free radical scavenging activity of 50% methanol extract.

The cluster analysis based on the fruit nutritional and phytochemical qualities grouped the cultivars into two main clusters: the honeydew melons in group I and the cantaloupe melons in group II (Figure 4). Furthermore, the principal component analysis revealed four principal components (PCs), each with an eigenvalue ≥ 1.00 (Table S1). The first four PCs cumulatively accounted for 85.15% of the total variation. Vitamin C, β-carotene and all the mineral elements (except calcium) contributed above 10% each to PC1, which accounted for 40.39% of the total variation (Table S2). In the PC2 that accounted for 20.88% of the total variation, flavonoids and free radical scavenging activity of the water extract contributed 28.88% and 36.81%, respectively (Table S2). Thus, a principal component biplot, based on the first two PCs that cumulatively accounted for 61.27% of the total variation, revealed cultivars with high associations with the measured fruit quality at harvest (Figure 5). All the nutritional and phytochemical traits were closely associated with the cantaloupe cultivars as indicated by their proximity to the vector line. In particular, cvs. Divine, Majestic, Cyclone, Magritte and Adore were closely associated with all the mineral elements evaluated, β-carotene and vitamin C. Cultivar E25F.00185 was closely associated with flavonoid, while cvs. E25F.00075 and Divine were closely associated with total phenolics. The two honeydew melons cvs. Honey Brew and Honey Star were not associated with any of the evaluated nutritional and phytochemical qualities.

### 2.2. Effect of Cultivar and Postharvest Storage on Fruit Nutritional and Phytochemical Quality

Cultivar and postharvest storage duration as individual factors, and interactively in some cases, significantly affected fruit quality, plus nutritional and phytochemical parameters (Table 3). Fruit flesh color a* and b* and total soluble solid (TSS) content were only affected by cultivar (Table 4). Cultivar MAB79001 had the highest color a* and TSS content. The minimum commercially acceptable TSS content for sweet melons is 9%; premium markets may require 11% or more [24]. Based on TSS content alone, all cultivars in this study were of satisfactory quality for local or international markets.

**Figure 6:**
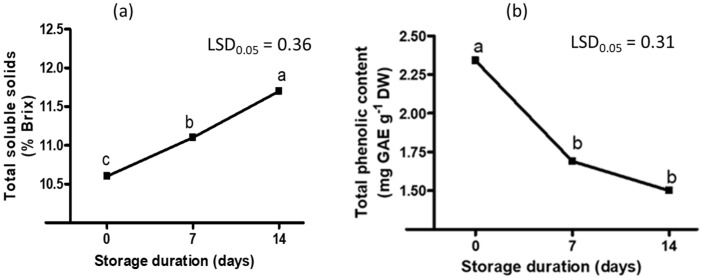
Effect of postharvest storage duration on sweet melon total soluble solids (**a**) and total phenolic content (**b**). Points on the line with different letters in each graph are significantly different at *p* ≤ 0.05 (Fisher’s protected LSD test, *n* = 3). GAE = gallic acid equivalent; DW = dry weight.

**Figure 7:**
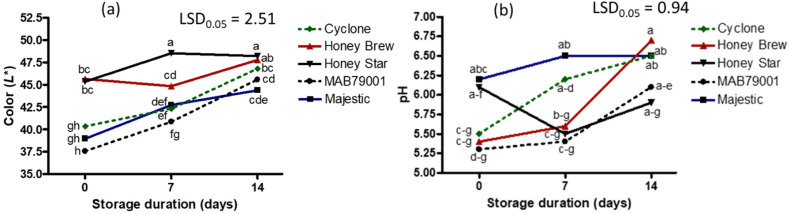
Interaction effect of sweet melon cultivars and storage duration on fruit color (**a**) and fruit juice pH (**b**). Points on lines with different letters in each graph are significantly different at *p* ≤ 0.05 (Fisher’s protected LSD test, *n* = 3). L* = lightness.

Postharvest storage duration also affected the TSS content (Table 3). The longer the storage duration, the higher the TSS (Figure 6). Thus, a 4% increase in TSS was recorded after 7 days of storage while it increased by 10% after 14 days of storage (Table S3). An increase in the TSS content has been reported in several fruit, which could be due to the alteration in cell wall structure, fruit climacteric nature and/or breakdown of complex carbohydrates into sucrose and other simple sugars including glucose and fructose [25,26,27,28,29]. An increase in postharvest storage duration, however, resulted in a decrease in total phenolic content (Figure 6, Table S3). Cultivar and postharvest storage duration did not affect electrical conductivity (average of 3.8 mS cm^−1^). On the other hand, cultivar and postharvest storage duration had a significant interaction effect on fruit color L*, fruit juice pH, vitamin C, β-carotene and flavonoid contents and free radical scavenging activity (Table 3). Fruit color L* and fruit juice pH of almost all cultivars increased over the postharvest storage duration (Figure 7). The cantaloupe cultivars particularly had a relatively high increase (≥14%) in fruit color L* after 14 days of storage in comparison to the honeydew cultivars (Table S4). The increase in the lightness of the fruit over the 14 days of storage indicates the absence of browning reactions [15]. The pH of a fruit has an inverse relationship with organic acids in the fruit juice. An increased pH in fruit during postharvest storage is accompanied by a decrease in organic acids due to hydrolysis, which subsequently increases the fruit sweetness and decreases sourness [30].

**Figure 8:**
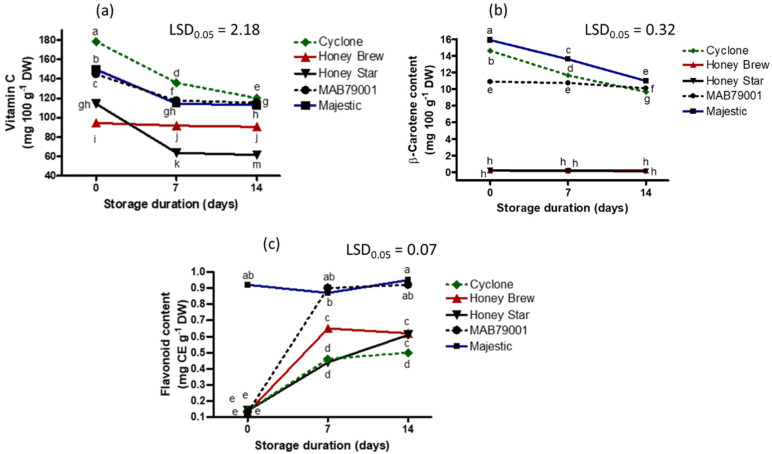
Interaction effect of sweet melon cultivars and storage duration on vitamin C (**a**), β-carotene (**b**) and flavonoid (**c**) contents. Points on lines with different letters in each graph are significantly different at *p* ≤ 0.05 (Fisher’s protected LSD test, *n* = 3). CE = catechin equivalent; DW = dry weight.

**Figure 9:**
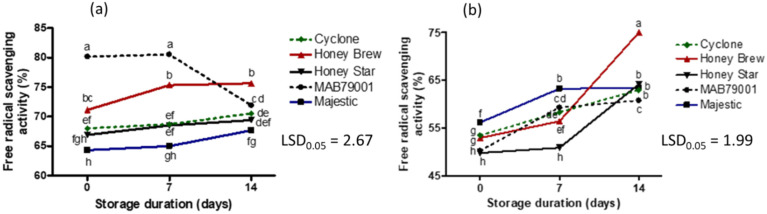
Interaction effect of sweet melon cultivars and storage duration on free radical scavenging activity of 50% methanol (**a**) and water (**b**) extracts. Points on lines with different letters in each graph are significantly different at *p* ≤ 0.05 (Fisher’s protected LSD test, *n* = 3).

All cultivars exhibited a decrease in vitamin C concentration with an increase in storage duration (Figure 8). At the end of the 14 days of storage, cv. Honey Star had the greatest decrease (46%) in vitamin C (Table S4). Cultivar Cyclone consistently had the highest vitamin C content during storage while cvs. Honey Brew and Honey Star had the lowest concentrations (Figure 8). A decline in vitamin C content of non-netted green-fleshed and orange-fleshed sweet melons collected from the field, and the non-netted green-fleshed Honey Brew, had the lowest concentration on day 17 of storage [14]. In the current study, an increase in storage duration resulted in a decline in the vitamin C content of sweet melons regardless of the cultivar type (Table S4). During postharvest storage, β-carotene concentration may remain stable, or decrease, depending on storage temperature [31]. In the current study, fruit were stored at the same temperature. A decrease in β-carotene content occurred in cantaloupe melons with an increase in postharvest storage duration (Figure 8, Table S4). The β-carotene concentration of cvs. Honey Star and Honey Brew, although the lowest, remained fairly stable during postharvest storage. Cultivar and flesh color influence β-carotene concentration among the sweet melon fruit while storage period and cultivar determine its retention. Unlike vitamin C and β-carotene concentrations, flavonoid content of fruit increased during storage in almost all cultivars (Figure 8, Table S4). Cultivar MAB79001 had the highest increase in flavonoid concentration (Table S4), which was 7-fold after 14 days of storage compared to its flavonoid concentration at harvest. With the exception of MAB79001 methanolic extract, there was an increase in antioxidant activity during storage of water and methanolic extracts of all cultivars (Figure 9, Table S4).

## 3. Materials and Methods

### 3.1. Effect of Cultivars on Fruit Nutritional and Phytochemical Quality at Harvest 

The procedures used to establish and maintain the plants from September 2016 until January 2017 were as described by Pulela et al. [10]. Ten fruit per cultivar per plot were randomly harvested at physiological maturity from sweet melon cultivars arranged in a randomized complete block design with four replicates (Figure S1). The cantaloupe cvs. Majestic, Magritte, Divine, Cyclone, MAB 79001, Adore, E25F.00075 and E25F.00185 and honeydew cvs. Honey Brew and Honey Star types were used [10]. The experiment was carried out in a non-temperature controlled, high plastic tunnel at the Agricultural Research Council—Vegetables, Industrial and Medicinal Plants, Roodeplaat, Pretoria, South Africa. The fruit were peeled with a sharp knife, sliced and homogenized. Fruit samples were kept at −80 °C, freeze-dried and ground into fine powders. The nutritional, phytochemical and antioxidant qualities of the fruit samples from each cultivar were analyzed as described below.

#### 3.1.1. Nutritional Analysis

Freeze-dried sweet melon samples were analyzed for phosphorus (P), potassium (K), calcium (Ca), magnesium (Mg) and zinc (Zn). The sample analysis was carried out with an aliquot of digested solution using inductively coupled plasma–optical emission spectrometry (ICP-OES) as described by Mahlangu et al. [32]. Each determination was in quadruplicate.

β-Carotene content was determined according to Moyo et al. [33]. Ice-cold hexane:acetone (1:1 *v*/*v*) was used to extract freeze-dried sweet melon samples. The pooled organic extract was gravity filtered through a 0.45 µm syringe filter before injection into an HPLC (Prominence-*i*-HPLC-PDA with LC-2030C sample cooler (Shimadzu, Kyoto, Japan)). There were four replicates for each determination at harvest, while analysis performed for postharvest experiment was carried out in triplicate.

Extraction and high-performance liquid chromatography (HPLC) quantification of vitamin C content were as described by Moyo et al. [33]. The extracted solution was gravity filtered through a 0.45 µm syringe filter before injection into an HPLC (Prominence-*i* HPLC-PDA, equipped with sample cooler LC-2030C (Shimadzu, Kyoto, Japan)). A calibration curve was plotted using L-ascorbic acid. Each determination was carried out in quadruplicate for analysis of fruit at harvest, while the analysis conducted for postharvest experiment was performed in triplicate.

#### 3.1.2. Phytochemical Analysis

Freeze-dried sweet melon samples (0.2 g) were extracted with 10 mL of 50% methanol in a sonication bath for 20 min. The mixtures were centrifuged and the supernatant was used for total phenolic and flavonoid analysis. The total phenolic content was determined using the Folin Ciocalteu colorimetric method [34]. Reaction mixes were incubated for 40 min at 25 °C and total phenolics content was determined by measuring absorbance at 725 nm, against a blank using a spectrophotometer (SPECORD^®^ 210 PLUS, Analytik Jena, Jena, Germany). Gallic acid (0.1 mg/mL^−1^) was used as a standard for preparing the calibration curve and the results were reported in mg gallic acid equivalent (GAE) per g dry weight. The assay was in quadruplicate for fruit analyzed at harvest, and triplicate for the postharvest experiment.

Flavonoid content quantification was carried out using the aluminum chloride method [35]. Absorbance was measured against a freshly prepared reagent blank and read at 510 nm using a spectrophotometer. Suitable aliquots of catechin (0.1 mg mL^−1^) were used to plot a standard calibration curve. The quantification was carried out in quadruplicate for fruit analyzed at harvest, and triplicates for the postharvest experiment. The results were presented in mg catechin equivalent (CE) per g dry weight.

#### 3.1.3. Free Radical Scavenging Activity

To determine free radical scavenging activity, freeze-dried, powdered samples were extracted with 50% methanol or distilled water at 20 mL g^−1^ in a sonication bath containing ice-cold water for 1 h. The 50% methanol extracts were concentrated *in vacuo* after vacuum filtration through Whatman No. 1 filter paper. Concentrated 50% methanol extracts were air-dried at 25 °C. Water extracts were vacuum filtered through Whatman No. 1 filter paper and lyophilized. Dried extracts (50% methanol and water extracts) were dissolved in methanol at a known sample concentration in glass vials to give a final assay concentration of 200 µg mL^−1^. Antioxidant activity was measured using the 2,2-diphenyl-1-picryl hydrazyl (DPPH) scavenging assay [36] with slight variations. The reaction mix contained 300 µL of each sample, 450 µL of methanol and 750 µL of DPPH solution (0.1 mM). The reaction mix was allowed to stand in the dark for 30 min at 25 °C after which absorbance at 517 nm was recorded. Ascorbic acid was used as a standard antioxidant (positive control) while methanol served as the negative control.

### 3.2. Effects of Cultivar and Postharvest Storage on Fruit Nutritional and Phytochemical Quality

Effects of cultivar and postharvest storage duration on fruit quality parameters and nutritional and phytochemical profile were determined with fruit harvested from cvs. Majestic, Cyclone and MAB 79001 (cantaloupe) and cvs. Honey Brew and Honey Star (honeydew) (Figure S2). Ten fruit per cultivar were selected. Fruit were packed into crates, arranged in a randomized complete block design with three replicates inside a cold room at 10 °C and approximately 95% relative humidity. Fruit were stored for 0, 7 and 14 days. At the end of each storage period, fruit were evaluated for vitamin C, β-carotene, total phenolic and flavonoid contents and free radical scavenging activity as described above. The following fruit quality parameters were also determined: fruit color chroma, total soluble solids (TSSs), pH and electrical conductivity (EC). Fruit flesh color was determined using a chromameter (CR400, Minolta Sensing Inc., Konica, Japan). The chromameter was calibrated with a standard white tile before measurements. Color changes were quantified in the L*, a* and b* color space. To determine TSS, pH and EC, fruit were sliced and blended to produce a puree. The puree was gravity filtered through a cheesecloth to produce sweet melon juice. The pH and EC of the juice were measured using a combo pH and EC meter (Hanna Instruments^®^ Inc., Curepipe, Mauritius). The TSS content of the sweet melon juice was determined using a hand-held refractometer (ATAGO, Tokyo, Japan) and expressed as % Brix.

### 3.3. Statistical Analysis

Data of fruit analysis at harvest were subjected to a one-way analysis of variance (ANOVA) using GenStat^®^ (ver. 11.1, VSN, Rothamsted, UK). Where significant differences were established, cultivar mean values were separated using Fisher’s protected least significant difference (LSD) test. To determine possible associations between measured parameters at harvest, data were subjected to Pearson correlation analysis using XLSTAT (ver. 17.04.36025 Add-in-soft, New York, NY, USA). Principal component and agglomerative hierarchical clustering analyses were also carried out to establish quality traits and cultivar associations. To determine the effects of cultivar and postharvest storage duration on physiochemical qualities and bioactive compounds of melons, data from the experiment involving postharvest duration and cultivars were subjected to a two-way analysis of variance using GenStat^®^. Significant interactions were used to explain results. Where interactions were not significant, mean values of main effects were separated using Fisher’s protected least significant difference test.

## 4. Conclusions

At harvest, the orange-fleshed sweet melon cultivars had higher mineral element (K, P, Mg, Ca and Zn), vitamin C and β-carotene contents than the green-fleshed cultivars. Thus, with some limitations, cultivar and fruit flesh color may be used as indicators of nutrient-rich sweet melons. Cultivar and postharvest storage duration had significant interaction effects on flavonoid, vitamin C and β-carotene concentrations, antioxidant activity, pH and color L* of the fruit. Total soluble solids, flavonoid content and antioxidant activity generally increased during postharvest storage, whereas the reverse was the case with vitamin C and β-carotene contents. Although some nutritional traits decreased as postharvest storage duration lengthened, sweet melon remains a comparatively healthy food in the human diet. Cultivars Cyclone and Majestic relatively outperformed other cultivars in terms of some postharvest storage nutritional qualities. In selecting the right cultivars, variation in nutritional traits of sweet melon cultivars as affected by postharvest storage should be an important consideration.

## Figures and Tables

**Table 1:** Sweet melon fruit mineral element content (mg kg^−1^ dry weight) at harvest.

Cultivar	Fruit Flesh Color	Potassium	Phosphorus	Magnesium	Calcium	Zinc
Adore	Orange	23,627 ± 262 b,c	1502 ± 23 c,d	1120 ± 20 b,c	657 ± 20 b	22 ± 1 a
Cyclone	Orange	24,646 ± 398 a,b	1674 ± 83 b,c	1191 ± 139 b	521 ± 39 c	23 ± 1 a
Divine	Orange	23,428 ± 778 b,c	1636 ± 61 b,c	1088 ± 20 b,c	405 ± 34 d	20 ± 1 a
E25F.00075	Orange	19,783 ± 594 e,f	1428 ± 65 d	998 ± 81 b,c,d	305 ± 15 e,f	22 ± 1 a
E25F.00185	Orange	23,651 ± 726 b,c	1646 ± 101 b,c	1050 ± 63 b,c	556 ± 36 c	21 ± 1 a
Honey Brew	Green	20,882 ± 138 d,e,f	1121 ± 13 e	809 ± 42 d,e	250 ± 28 f	13 ± 0 c
Honey Star	Green	19,488 ± 781 f	1213 ± 23 e	717 ± 32 e	323 ± 23 e,f	17 ± 2 b
MAB 79001	Orange	22,227 ± 383 c,d	1703 ± 87 a,b	921 ± 10 c,d,e	352 ± 11 d,e	22 ± 2 a
Magritte	Orange	25,290 ± 182 a	1873 ± 25 a	1015 ± 60 b,c,d	309 ± 13 e,f	23 ± 1 a
Majestic	Orange	21,266 ± 307 d,e	1696 ± 50 a,b	1495 ± 110 a	733 ± 13 a	22 ± 1 a
LSD_0.05_		1537	185	207	73	3

Values (±standard error of means) in the same column followed by the same letters are not significantly different at *p* ≤ 0.05 (Fisher’s protected LSD test, *n* = 4).

**Table 2:** Pearson correlation of nutritional and phytochemical traits of cantaloupe and honeydew melon cultivars at harvest.

Variable	TPC	Flav	Vit. C	β-Car	K	P	Mg	Ca	Zn	FSA (W)
Flavonoids	−0.162									
Vitamin C	0.236	**0.439** ^a^								
β-Carotene	0.051	0.008	0.184							
K	−0.270	0.229	0.343	**0.524**						
P	−0.042	0.183	**0.387**	**0.825**	**0.679**					
Mg	0.249	0.055	0.260	**0.538**	0.238	**0.572**				
Ca	0.204	0.267	**0.417**	0.228	0.273	**0.370**	**0.761**			
Zn	0.175	0.129	**0.699**	**0.591**	**0.437**	**0.691**	**0.457**	**0.467**		
FSA (W)	0.229	−**0.919**	−0.309	−0.009	−0.240	−0.059	0.192	−0.090	−0.045	
FSA (AM)	0.043	0.011	0.022	−**0.391**	0.066	−0.184	−0.192	0.134	−0.035	−0.124

^a^ Values in bold indicate significant (*p* = 0.05) correlation between 2 parameters. TPC = total phenolics content; Flav = flavonoid; Vit. C = vitamin C; β-Car = β-carotene; K = potassium; P = phosphorus; Mg = magnesium; Ca = calcium; Zn = zinc; FSA (W) = free radical scavenging activity of water extract; FSA (AM) = free radical scavenging activity of 50% methanol extract.

**Table 3:** Analysis of variance for the effect of cultivar and storage duration on sweet melon fruit quality parameters, plus nutritional and phytochemical contents.

Source	df	Mean Squares										
		TPC	Flav	Vit. C	β-Car	FSA		Color			TSS	pH
						W	AM	L*	a*	b*		
Rep	2	0.061	0.001	3.345	0.028	1.70	4.43	5.54	0.70	62.14	0.05	0.50
C	4	0.542 ns	0.466 **	6435.795 **	387.061 **	68.25 **	222.26 **	61.85 **	488.75 **	33.77 *	3.64 **	0.85 ns
S	2	2.927**	0.807 **	5836.557 **	17.369 **	617.22 **	3.48 ns	93.32 **	1.23 ns	23.72 ns	4.29 **	1.78 **
C × S	8	0.134 ns	0.085 **	417.577 **	5.009 **	53.74 **	22.18 **	6.96 *	1.47 ns	2.95 ns	0.44 ns	0.41 *
Error	20	0.171	0.000	1.168	0.025	1.68	3.01	2.57	2.24	24.03	0.22	0.11
Total	44											

ns, *, ** = not significant, or significant at *p* < 0.05 or *p* < 0.001, respectively. Rep = replicate; C = cultivar; S = storage; TPC = total phenolics content; Flav = flavonoid; Vit. C = vitamin C; β-Car = β-carotene; FSA = free radical scavenging activity; W = water extract; AM = aqueous methanol extract; TSS = total soluble solids; L* = lightness; a* = red/green; b* = blue/yellow.

**Table 4:** Effect of cultivar on flesh color and total soluble solids of melon juice.

Cultivar	Color		Total Soluble Solids (% Brix)
	a*	b*	
Cyclone	7.3 ± 0.7 b	13.4 ± 2.8 a,b	10.1 ± 0.3 c
Honey Brew	−5.4 ± 0.8 c	11.1 ± 1.6 b	11.4 ± 0.3 b
Honey Star	−5.8 ± 0.7 c	12.1 ± 1.5 b	11.2 ± 0.3 b
MAB79001	9.3 ± 0.7 a	10.9 ± 1.8 b	11.8 ± 0.5 a
Majestic	6.7 ± 1.1 b	15.6 ± 3.8 a	11.2 ± 0.4 b
LSD_0.05_	1.6	2.8	0.3

Values (±standard error of means) in each column followed by different letters are significantly different at *p* ≤ 0.05 (Fisher’s protected LSD test, *n* = 3); a* = red/green; b* = blue/yellow.

## Data Availability

Not applicable.

## Supplementary Materials

The following supporting information can be downloaded at: https://www.mdpi.com/article/10.3390/plants11162136/s1, Figure S1: Experimental layout of sweet melon cultivar trial in a non-temperature controlled plastic tunnel; Figure S2: Sweet melon cultivars used for postharvest storage; Table S1: Eigenvectors from principal component (PC) analysis based on the nutritional and phytochemical quality at harvest; Table S2: Contribution (%) of each variable to the principal components; Table S3: Changes (%) in total soluble solid and total phenolic contents as influenced by postharvest storage duration; Table S4: Changes (%) in fruit quality parameters, nutritional and phytochemical contents as influenced by postharvest storage duration.
