# Supplementary material for: Cultivar and Postharvest Storage Duration Influence Fruit Quality, Nutritional and Phytochemical Profiles of Soilless-Grown Cantaloupe and Honeydew Melons
Source: Plants (Basel). 2022 Aug 17;11(16):2136. doi: 10.3390/plants11162136 (PMC9413578; doi:10.3390/plants11162136)
Supplement: Supplementary file 1 [file plants-11-02136-s001.zip › plants-1864464-supplementary.pdf]

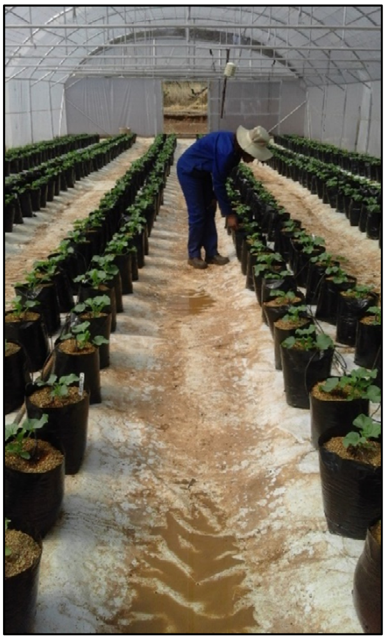

Figure S1: Experimental layout of sweet melon cultivar trial in a non-temperature controlled plastic tunnel

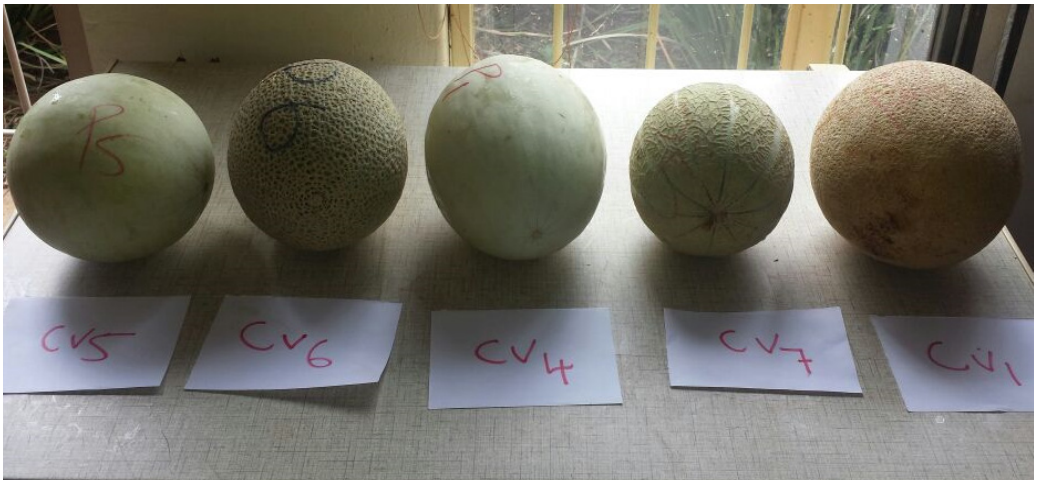

Figure S2: Sweet melon cultivars used for postharvest storage. CV1 = Majestic; CV4 = Honey Brew; CV5 = Honey Star; CV6 = Cyclone; CV7 = MAB79001.

Table S1. Eigenvectors from principal component (PC) analysis based on the nutritional and phytochemical quality at harvest.

Traits	PC1	PC2	PC3	PC4
K	0.334	-0.174	-0.285	0.375
P	0.430	0.016	-0.284	0.103
Mg	0.371	0.271	0.170	-0.055
Ca	0.308	0.042	0.388	0.106
Zn	0.420	0.104	0.012	0.056
β-Carotene	0.371	0.181	-0.371	-0.100
Vitamin C	0.320	-0.110	0.390	-0.060
Total phenolics	0.050	0.374	0.504	-0.129
Flavonoids	0.173	-0.537	0.187	-0.338
FSA(M)	-0.074	-0.204	0.280	0.808
FSA(W)	-0.130	0.607	-0.048	0.183
Eigenvalue	4.443	2.296	1.549	1.078
Variability (%)	40.394	20.877	14.081	9.796
Cumulative variability (%)	40.394	61.271	75.352	85.148

K = potassium; P = phosphorus; Mg = magnesium; Ca = calcium; Zn = zinc; FSA (W) = free radical scavenging activity of water extract; FSA (M) = free radical scavenging activity of 50% methanol extract

Table S2. Contribution (%) of each variable to the principal components.

Traits	PC1	PC2	PC3	PC4
K	11.184	3.029	8.103	14.049
P	18.474	0.025	8.056	1.051
Mg	13.730	7.350	2.886	0.305
Ca	9.465	0.178	15.049	1.116
Zn	17.674	1.085	0.013	0.313
β-Carotene	13.781	3.285	13.771	1.010
Vitamin C	10.215	1.216	15.179	0.364
Total phenolics	0.253	13.973	25.357	1.654
Flavonoids	3.002	28.881	3.501	11.458
FSA(M)	0.542	4.170	7.859	65.326
FSA(W)	1.679	36.809	0.226	3.353

K = potassium; P = phosphorus; Mg = magnesium; Ca = calcium; Zn = zinc; FSA (W) = free radical scavenging activity of water extract; FSA (M) = free radical scavenging activity of 50% methanol extract

Table S3. Changes (%) in total soluble solid and total phenolic contents as influenced by postharvest storage duration.

Postharvest storage duration	Total soluble solid	Total phenolic content
7 days	4.2	-27.8
14 days	10.0	-36.1

Positive and negative values indicate percentage increases and decreases, respectively.

Table S4. Changes (%) in fruit quality parameters, nutritional and phytochemical contents as influenced by postharvest storage duration.

	Flavonoid		Vitamin C		β-Carotene		FSA (W)		FSA (M)		Colour *L**		pH	
Cultivar	7 d	14 d	7 d	14 d	7 d	14 d	7 d	14 d	7 d	14 d	7 d	14 d	7 d	14 d
Cyclone	231.9	258.4	-23.8	-32.7	-20.2	-33.9	9.0	17.7	1.0	3.7	4.9	16.0	13.8	18.6
Honey Brew	382.0	362.9	-2.9	-4.2	0.0	-1.4	6.6	41.5	1.9	2.0	-1.8	4.7	4.9	25.8
Honey Star	212.1	328.2	-44.2	-46.0	-10.8	-42.5	2.2	28.9	2.4	3.7	7.0	6.3	-9.6	-3.1
MAB79001	600.6	611.0	-18.8	-20.2	-1.4	-7.3	18.1	21.1	0.5	-10.3	8.8	21.4	2.4	15.9
Majestic	-5.1	3.6	-23.4	-24.6	-14.6	-31.0	12.6	12.8	1.0	5.2	9.7	13.9	4.3	4.2

Positive and negative values indicate percentage increases and decreases, respectively. FSA (W) = free radical scavenging activity of water extract; FSA (M) = free radical scavenging activity of 50% methanol extract; d = days.
